# Support Vector Machine Weather Prediction Technology Based on the Improved Quantum Optimization Algorithm

**DOI:** 10.1155/2021/6653659

**Published:** 2021-04-13

**Authors:** Jinlei Zhang, Xue Qiu, Xiang Li, Zhijie Huang, Mingqiu Wu, Yumin Dong

**Affiliations:** College of Computer and Information Science, Chongqing Normal University, Chongqing, China

## Abstract

Emotion recognition is a research hotspot in the field of artificial intelligence. If the human-computer interaction system can sense human emotion and express emotion, it will make the interaction between the robot and human more natural. In this paper, a multimodal emotion recognition model based on many-objective optimization algorithm is proposed for the first time. The model integrates voice information and facial information and can simultaneously optimize the accuracy and uniformity of recognition. This paper compares the emotion recognition algorithm based on many-objective algorithm optimization with the single-modal emotion recognition model proposed in this paper and the ISMS_ALA model proposed by recent related research. The experimental results show that compared with the single-mode emotion recognition, the proposed model has a great improvement in each evaluation index. At the same time, the accuracy of emotion recognition is 2.88% higher than that of the ISMS_ALA model. The experimental results show that the many-objective optimization algorithm can effectively improve the performance of the multimodal emotion recognition model.

## 1. Introduction

It is worth mentioning that rainfall is the only input element source for the hydrologic cycle. However, excessive rainfall and the scarcity of it on Earth affect the tremendous flooding and severe drought that occur over short and long intervals [1]. Therefore, rainfall forecasting can prevent many natural disasters, thereby saving human lives. The accuracy of rainfall forecasting can also help the preparation of efficient structural and nonstructural designs for disaster management. Weather forecast (measurement) [2–7] is to use modern science and technology to predict the state of the Earth's atmosphere at a certain place in the future. Since prehistory, human beings have begun to predict the weather to arrange their work and life accordingly (such as agricultural production and military operations). Today's weather forecast mainly uses the collection of a large number of data (temperature, humidity, wind direction and wind speed, air pressure, etc.), and then uses the current understanding of atmospheric processes (meteorology) to determine future air changes. Because of the disorder of atmospheric process and the fact that science does not know it thoroughly, there are always some errors in weather forecast.

Weather forecast can be divided into three types: short-term weather forecast [8, 9] (2-3 days), medium-term weather forecast (4–9 days), and long-term weather forecast [10, 11] (more than 10–15 days). This study will focus on short-term weather forecast. According to the coverage area, the weather forecast can be divided into large-scale forecast (generally referring to the forecast of a continent or country), medium-scale forecast (usually referring to the forecast of a province (region), state, and region), and small-scale forecast (such as the forecast of a county, city, etc.). The medium-scale city forecast is carried out in this study. Research studies on hybrid techniques using data-driven models, such as artificial neural networks (ANNs), genetic programming (GP), and adaptive neurofuzzy inference systems (ANFISs), integrated with different optimization methods (e.g., particle swarm optimization (PSO), genetic algorithm (GA), and differential evolution (DE) algorithm) [12] have been published over the past decades and demonstrated positive outcomes for solving hydrology and water resource problems, such as rainfall runoff, river stage, evaporation, sediment, and groundwater. Abrahart et al. [13] used a pruning algorithm (PA) and a genetic algorithm (GA) to optimize data-driven models for runoff forecasting. Yaseen et al. [14] proposed the novel hybrid data-intelligence model for forecasting monthly rainfall with uncertainty analysis. Chau [15] applied a particle swarm optimization (PSO) algorithm to train ANNs for river stage prediction. He found that PSO could optimize the applied ANNs. In recent years, many new metaheuristics have been proposed. For example, Gai-Ge Wang proposed the moth search algorithm (MSA) [16] in 2018, and many researchers used the algorithm for optimization tasks in various fields. Then, Wang et al. proposed monarch butterfly optimization (MBO) [17] in 2019. Ali Heidari et al. proposed Harris hawk optimization (HHO) [18] in 2019 and achieved good results. Li et al. proposed the slime mould algorithm (SMA) [19] in 2020 and achieved good optimization results. Then, Moreno-Pineda et al. proposed the improved dragonfly algorithm (DA) [20]; the method can be used as a useful, auxiliary tool for solving complex optimization problems. Next, Yu et al. proposed a moth flame optimizer [21], which has achieved remarkable results in solving complex optimization problems.

Based on the above analysis, a new weather prediction model based on the improved quantum genetic algorithm (IQGA) [22] and support vector machines (SVMs) [23–25] is proposed to solve the problems in short-term and medium-range weather prediction. In real life, the prediction of weather forecast is a complicated process. It is difficult for people to find the rule and use it to predict the trend of future weather facing the complicated data. In this study, real weather data with 140,000 rows and 21 data features are collected from Kaggle (https://www.kaggle.com/jsphyg/weather-dataset-rattle-package). Since the original dataset is collected and recorded by weather stations through rain radars and satellite images, the data must be analyzed and processed before establishing the weather prediction model. After dataset processing, SVM in machine learning algorithm is used to model, and QGA is used to optimize the parameters to maximize the efficiency of the model. There are 21 features and 1 tag in 22 columns of data features. It is difficult to find the rules contained in the data through visual observation. However, SVM algorithm is very powerful; it can do data mining well to achieve better classification effect. However, one of its disadvantages is that it is greatly influenced by the parameters. Because many parameters will affect the efficiency of the algorithms at the same time, it is very inefficient to use traditional grid search, learning curve, and other parameter adjustment methods. It will take a lot of time and often cannot achieve the optimal classification effect. At this time, the efficiency and optimization advantages of quantum genetic algorithm can maximize the efficiency of the algorithms. The performance comparison between the model obtained by the original parameter adjustment method and the model optimized by the quantum genetic algorithm in the test set shows that the model optimized by the quantum genetic algorithm has a significant improvement in the prediction accuracy, AUC, and other test indicators. It is proved that the method is effective for determining optimization parameters and improving machine learning efficiency. The improved quantum genetic algorithm can be applied to the parameter optimization of other machine learning algorithms to increase the calculation efficiency and the optimization accuracy. It can also be used in various optimization problems to improve the search efficiency.

## 2. Related Technology Introduction

### 2.1. Preprocessing of Weather Data

As far as the current social development is concerned, we are all in the background of big data, and we cannot live without the influence of the data system. In the fierce competition process of all walks of life, the results of data mining often affect the decision-making direction of the whole project and then affect the final implementation and development results. It can be seen that data mining has become the focus of attention, and the data preprocessing which plays an important role in the process of data mining has also been further studied and found. Data preprocessing [26, 27] is the first and most important step in data mining in this study, and we must ensure the accuracy and validity of weather data at the beginning of data mining. Only by doing a good job of data preprocessing and further improving the quality of data contained in the database can we fundamentally mine valuable data and improve the generalization ability of the model.

The complex high-dimensional dataset used in this study includes the daily weather forecast of many Australian meteorological stations in 10 years. There are about 140,000 pieces of data points, each of which has 22 characteristics, 21 columns are characteristic matrices (including time, place, maximum temperature of the day, rainfall, and wind speed), and the last column is tag (indicating whether it will rain tomorrow). In order to forecast the weather, we need to build the model forecast tag in the 21-column feature. The feature matrix of the dataset is composed of a part of classified variables and a part of continuous variables. For example, although the degree of cloud cover is expressed by numbers, it is a classified variable in essence. Most of the features are collected natural data, such as evaporation, sunshine time, and humidity, while a few of the features are composed of humans. There are also variables that simply represent sample information, such as the location and time of collection.

In the data preprocessing of this study, we will divide the training set and the test set first and then start the data preprocessing. This is because the test set is often impossible to get in advance in reality, and we do not want any process of our modeling to be affected by the test set data; otherwise, it is equivalent to telling part of the predicted answers of the model in advance. In this study, all operations we performed on the training set must be performed on the test set once.

After obtaining the complex real weather dataset, we first observe the composition of the dataset as shown in Table 1 and then separate the training set and the test set, as well as the features and tag.

By observing the aforementioned dataset, we can find that there are a lot of empty data in the dataset, and there is also a case of dimensional inconsistency. At the same time, the data are composed of classification variables and continuous variables which need our data processing.

**Figure 1:**
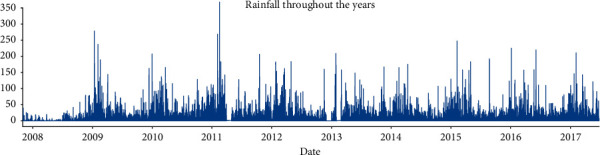
Year rainfall trend.

**Figure 2:**
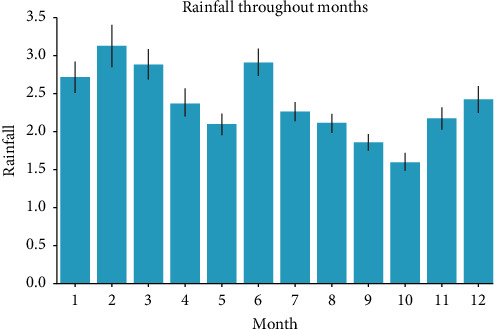
Monthly rainfall trend.

**Figure 3:**
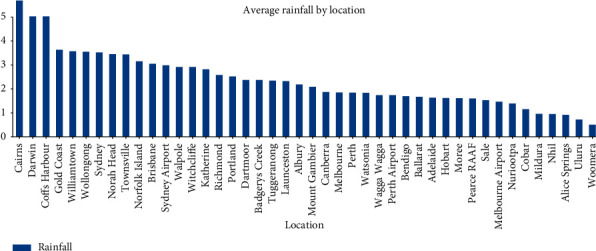
Location rainfall trend.

In this study, we first process the characteristics of the data. By observing the data, we can know that all data include 21 characteristics: time, place, temperature, humidity, cloud cover, rainfall, etc. They also include our tag: whether it will rain tomorrow. Through data visualization [28, 29] (Figure 1 (year rainfall trend chart), Figure 2 (monthly rainfall trend chart), and Figure 3 (location rainfall trend chart)), we can find that time, location, and other characteristics will affect our tag.

Through data visualization, we can intuitively find that the year has little impact on the tag, but it will increase the complexity of the data, but the month will have a great impact on the tag, and the location has a very obvious impact on the tag. In this way, these data can help the model to make a better and more accurate judgment on the label. So, we cannot delete it directly; we need to deal with it. The following ingenious processing of weather data is made in this study.

**Figure 4:**
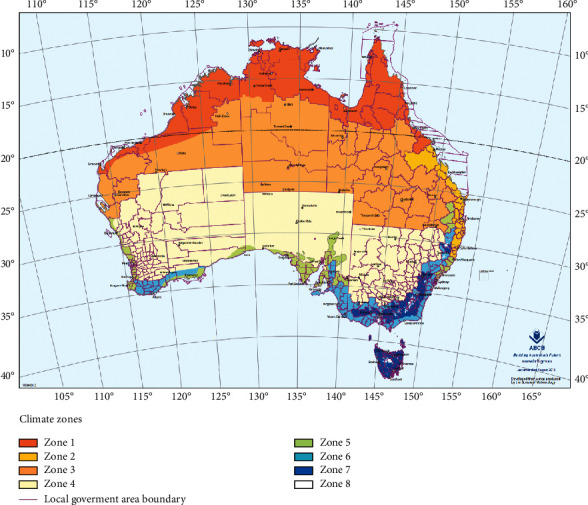
City climate map of Australia.

1. The month in time is extracted; because through data visualization analysis and common sense, we can know that the month will affect the probability of rain.
2. Does the day in time affect the tag? On the surface, it may not have any effect, but if it rains today, will the probability of rain tomorrow increase? Thinking of this, We have added new features to the dataset about whether it is raining today. We can set the threshold value by using today's precipitation. In this study, we set rainfall greater than 1 mm as today's rain and set the rest as no rain. After treatment, we add a new feature of whether it rains today.
3. We know that the climate corresponding to the location will affect our tag, so this study cleverly corresponds the location to the local climate, and we found the Australian land as shown in Figure 4 on Google.

This is produced by the Australian Meteorological Agency and the Australian Building Code Board (ABCB), which divides the climate regions of different cities in different regions of Australia. The distribution map is divided into eight regions, which is very suitable for us to classify. If we can transform 49 locations into eight different climates, this information should be useful for judging whether it rains or not. Based on the data of the Meteorological Bureau and ABCB, we have made the corresponding climate-type data of the main cities in Australia and saved them in the csv-1 file, but we found that the location in the original weather dataset does not represent the city but the name of the weather station. We need to convert the name of the weather station in the dataset into the name of the city. We use the shortest direct distance between two points to convert. Next, we do a web crawler [30, 31] on Google to get the longitude and latitude of each city and save them in data csv-2. Next, we get the latitude and longitude corresponding to all weather stations in the dataset through the web crawler and save them in the csv-3 file. Next, we calculate the distance from the weather station to each city, find the city closest to the nearest weather station, and convert the location in the dataset into the city name closest to it. In this way, we can successfully convert the name of the climate station into the city name, and then we can use the csv-1 climate file to successfully convert each city name into the corresponding climate.

After finishing the above processing, we will fill in the missing value, code the type variable, and make the continuous variable dimensionless. Since our eigenmatrix is composed of two types of data, type and continuous, we must adopt different strategies to fill the missing values. In this study, if the feature is classified, we use mode to fill. If the feature is continuous, we use mean to fill. At this time, because we have divided the training set and the test set, because our test set may not be many pieces of data in reality, may be our test set has only one piece of data, and a certain feature is null, at this time, the mode of the test set itself does not exist, so in order to avoid this situation, we usually assume that the data distribution and properties of the test set and training set are similar, so we use the mode and mean of the training set to fill the test set. The normalization method is used in the dimensionless treatment of continuous variables.

After completing the above operations, our data preprocessing work is finished completely, and then we start to model.

### 2.2. Improved Quantum Genetic Algorithm

Quantum genetic algorithm (QGA) [32–34] is a new developed probability optimization method based on the concept and theory of quantum computing in recent years. Compared with the classical genetic algorithm, QGA has great differences in many aspects. The chromosome of quantum genetic algorithm is not represented by a definite value (such as binary number, floating-point number, or symbol), but by qubit or random probability. Correspondingly, the state of the chromosome is not a definite state, but a superposition state. The main way of chromosome evolution of quantum genetic algorithm is not the selection, crossover, and mutation of genetic algorithm, but the quantum. In addition, the crossover and mutation operations introduced in quantum genetic algorithm are different from the crossover and mutation of genetic algorithm in the way of implementation. These ingenious designs make quantum genetic algorithm have better population diversity and parallel computing ability.

#### 2.2.1. Quantum Bit Coding

The smallest information unit in QGA is represented by qubits. A qubit can not only represent two states of 0 and 1 but also represent any superposition state between them. Therefore, the state of a qubit can be expressed as *φ*_*i*_=*α*_*i*_|0〉+*β*_*i*_|1〉, where *α* and *β* are probability amplitudes of 0 and 1, respectively, and the following normalization conditions are met: |*α*|^2^+|*β*|^2^=1, |*α*|^2^ represents the probability that the observed value of the quantum state is 0, and |*β*|^2^ represents the probability that the observed value of the quantum state is 1.

Therefore, a qubit can store and express information of two states at the same time. A qubit string with a length of *N* can store and express 2^*n*^ different solutions at the same time. The encoded chromosomes are as follows:

(1) $$\begin{array}{c} p_{j}^{t}=\left[\begin{array}{c} \alpha_{1}^{t}\\ \beta_{1}^{t} \end{array}{\left|\begin{array}{c} \alpha_{2}^{t}\\ \beta_{2}^{t} \end{array}\right|}_{\ldots }^{\ldots }|\begin{array}{c} \alpha_{m}^{t}\\ \beta_{m}^{t} \end{array}\right], \end{array}$$

where *p*_*j*_^*t*^ is the chromosome of the *j* th individual of the *t* generation and *m* is the quantum bit number of chromosomes.

#### 2.2.2. Quantum

The evolution of quantum genetic algorithm is to use the quantum gate to rotate the quantum state, which is another significant difference from classical genetic algorithm. The transformation of the quantum logic gate is unitary transformation in the Hilbert space, which can ensure that the superposition state after change still meets the normalization condition.

In the quantum genetic algorithm, the quantum rotating gate [35] is used to update the individuals in the population. The matrix expression is as follows:

(2) $$\begin{array}{c} R\left(\theta \right)=\left(\begin{array}{cc} \cos\left(\theta \right) & -\sin\left(\theta \right)\\ \sin\left(\theta \right) & \cos\left(\theta \right) \end{array}\right), \end{array}$$

where *θ* is the angle of rotation. For any quantum superposition state *φ*_*i*_=*α*_*i*_|0〉+*β*_*i*_|1〉, the update operation is expressed as follows:

(3) $$\begin{array}{c} \left(\begin{array}{c} \alpha_{i}^{\prime }\\ \beta_{i}^{\prime } \end{array}\right)=R\left(\theta \right)\left(\begin{array}{c} \alpha_{i}\\ \beta_{i} \end{array}\right)=\left(\begin{array}{cc} \cos\left(\theta_{i}\right) & -\sin\left(\theta_{i}\right)\\ \sin\left(\theta_{i}\right) & \cos\left(\theta_{i}\right) \end{array}\right)\left(\begin{array}{c} \alpha_{i}\\ \beta_{i} \end{array}\right). \end{array}$$

The rules for *θ*_*i*_ are as follows:

(4) $$\begin{array}{c} \theta_{i}=S\left(\alpha_{i},\beta_{i}\right)\Delta \theta_{i}. \end{array}$$

Among them, (*α*_*i*_, *β*_*i*_) represents the probability amplitude of the *i*th qubit of the chromosome. *S*(*α*_*i*_, *β*_*i*_) and Δ*θ*_*i*_ are the rotation direction and rotation angle, respectively, which are selected according to the predetermined adjustment strategy.

**Figure 5:**
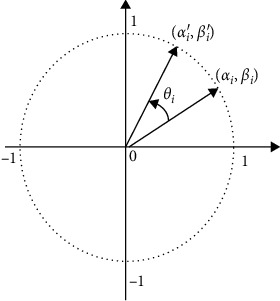
Diagram of rotation variation.

Figure 5 is the diagram of rotation variation. Under the guidance of the *i* position of the optimal solution chromosome, the current solution chromosome rotates clockwise to increase *α*_*i*_^*t*^ to *α*_*i*_^*t*+1^ and decrease *β*_*i*_^*t*^ to *β*_*i*_^*t*+1^. It evolves in the optimal direction after rotation variation.

The quantum rotation gate completes the update operation by changing the amplitude of the quantum superposition state. The change of the amplitude changes the probability of each basic state obtained after measurement while maintaining the diversity of the population. The initial population will gradually deflect to the species with higher adaptability after the quantum rotation gate operation and finally converge to the optimal population to find the optimal solution of the problem.

#### 2.2.3. Improved Quantum Genetic Algorithm

In this paper, the traditional quantum genetic algorithm is improved in three aspects, and an improved quantum genetic algorithm is proposed. Next, three improvements are introduced.

(1) Improvement of the quantum population initialization adjustment strategy: quantum genetic algorithm uses the quantum superposition state to represent individuals in the population. Therefore, compared with the classical genetic algorithm, it maintains the same population diversity and greatly reduces the population size. In general, the population initialization of quantum genetic algorithm uses formulas to encode individuals, and each gene collapses to |0〉 and |1〉 with equal probability which can ensure that the initial population is randomly distributed in the global solution space. However, this may cause that a local solution space is not searched or falls into the local optimal solution, and the purpose of optimization cannot be completed quickly.

In order to make the initial population more evenly spread over the whole solution space, the initial population can be initialized by grouping. When the traditional quantum genetic algorithm uses the formula to encode the quantum state, the parameter *θ* belongs to the interval [0,2*π*], that is, the quantum state can be located anywhere on the unit circle in the two-dimensional space, and finally converges to one point of ±*|*|0〉 or ±|1〉 through the action of multiple quantum rotation gates.

Therefore, in this study, the initial population is divided into five subpopulations, and the population initialization rules are as follows:

(5) $$\begin{array}{c} \text{Angle}\left(c\_p\right)=\left\{\begin{array}{c} \frac{\pi *0.05*p\_s}{2\left(p\_s+1\right)},\;\text{if }c\_p\leq 0.05*p\_s,\\ \frac{\pi *0.25*p\_s}{2\left(p\_s+1\right)},\;\text{if }0.05*p\_s<c\_p\&\&c\_p\leq 0.25*p\_s,\\ \frac{\pi *0.5*p\_s}{2\left(p\_s+1\right)},\;\text{if }0.25*p\_s<c\_p\&\&c\_p\leq 0.75*p\_s,\\ \frac{\pi *0.75*p\_s}{2\left(p\_s+1\right)},\;\text{if }0.75*p\_s<c\_p\&\&c\_p\leq 0.95*p\_s,\\ \frac{\pi *0.95*p\_s}{2\left(p\_s+1\right)},\;\text{if }0.95*p\_s\leq c\_p, \end{array}\right) \end{array}$$

where Angle is the initialization angle of the current population, *p*_*s* is the population size, and *c*_*p* represents the currently initializing individual label.

The above formula divides the initial population into five parts so that the initial population can spread over the whole solution space. *π*/2 is selected for the initialization of multiple groups of population in the appeal formula but not for the division of the 2*π* interval because if the 2*π* interval is divided, it is possible to make the initialized population fall on a certain coordinate axis, resulting in the amplitude of the basic state |0〉 or |1〉 being 0. Thus, the final observation result is a certain value which does not meet the randomness condition.

This initialization method can make the initial population spread over the whole solution space and avoid the disadvantage that the probability amplitude of the quantum basic state is too average which may result in the measurement results concentrated in a certain interval. It can search from multiple local solution spaces at the same time, ensure the diversity of chromosomes, and improve the convergence rate of the algorithm.

(2) Improvement of the rotation angle adjustment strategy: the core of quantum genetic algorithm is the operation of update evolution which uses quantum rotation gate to rotate the quantum state at a certain angle. Premature convergence may occur when the rotation angle is too small; the convergence rate is too slow when the rotation angle is too large. Therefore, how to choose the rotation angle of the quantum rotary gate will directly affect whether the quantum genetic algorithm can find the optimal solution quickly and effectively. However, the rotation angle of the quantum rotation gate selected by the update strategy is usually fixed in the standard quantum genetic algorithm, or the evolution operation is completed according to the update strategy table. The two rotation angles are relatively fixed, which makes the algorithm not flexible enough to play the best performance of the quantum genetic algorithm.

Our improved quantum genetic algorithm uses a dynamic adjustment method for the rotation angle of the quantum rotation gate. When the current number of iterations is small, it indicates that there is still a large optimization space for the current quantum individual as a whole, and large angle rotation is required to achieve faster convergence. When the current number of iterations is closer to the maximum number of iterations, it shows that the whole quantum individual has become more and more excellent, so we only need to gradually reduce the rotation angle to find the best individual.

The dynamic adjustment strategy for the rotation angle *θ*_new is as follows:

(6) $$\begin{array}{c} \theta \_\text{new}=\frac{\text{iter}\_\text{sum}-\text{iter}\_\text{cur}}{\text{iter}\_\text{sum}}*\theta \_\max. \end{array}$$

Among them, *θ*_max is the maximum value of the set rotation angle. iter_sum is the maximum number of iterations set. iter_cur is the number of iterations currently carried out. This adjustment method can associate the rotation angle with the number of iterations and dynamically adjust the rotation angle, so as to improve the convergence rate of the whole body. It can also make the current individuals to be updated always and rotate faster towards the better individuals so that the algorithm can converge to the optimal solution faster. The quantum rotation gate strategy is shown in Table 2.

(3) Selection strategy improvement: the new mechanism of probability selection is adopted to abandon the traditional strategy of keeping excellent individuals and discarding individuals with low adaptability, and the probability of being selected can be determined flexibly according to the size of individual adaptability, which not only ensures the population richness but also effectively optimizes the algorithm into the local optimal solution, and each selection makes the algorithm move towards a good evolutionary direction. The specific implementation form is as follows:

(7) $$\begin{array}{c} p=\frac{f\_\text{value}}{\text{sum}\_\text{value}}, \end{array}$$

where *p* is the current individual retention probability, *f*_value is the current individual fitness value, and sum_value is the total individual fitness value of the population. The strategy ensures that no matter whether the individual is good or bad, there is a chance to keep it. At the same time, the higher the fitness, the greater the probability of keeping it, so the algorithm can optimize the population every time.

#### 2.2.4. Support Vector Machine

Support vector machine [36–38] (SVM, also known as support vector network) is the most concerned algorithm in machine learning. It comes from statistical learning theory. From the practical application, SVM is very good in all kinds of practical problems. It is widely used in handwritten digit recognition and face recognition and plays an important role in text and hypertext classification because SVM can greatly reduce the needs of standard inductive and transductive settings for marker training examples. At the same time, SVM is also used to perform image classification and image segmentation system. Experimental results show that, after only three or four rounds of correlation feedback, SVM can achieve much higher search accuracy than the traditional query refinement schemes. In addition, biology and many other sciences are the favourites of the SVM. SVM has been widely used in protein classification, and the industry average level of the compound classification can reach more than 90% accuracy. In the cutting-edge research of biological science, support vector machine is also used to identify various features used for model prediction, so as to find out the influencing factors of various gene expression results. From the academic point of view, SVM is a machine learning algorithm close to deep learning. Linear SVM can be regarded as a single neuron of neural network (although loss function is different from the neural network), while nonlinear SVM is equivalent to a two-layer neural network. If multiple kernel functions are added to the nonlinear SVM, multilayer neural network can be imitated.

SVM is developed from the optimal classification surface in the case of linear separability. The basic idea of the SVM is to map the input vector to a high-dimensional feature space through a preselected nonlinear mapping and turn the required nonlinear problem into a linear separable problem in the high-dimensional space. In this high-dimensional space, an optimal linear classification surface is constructed which can separate the two types. Moreover, the classification interval between the two categories is maximized.

Set the sample set to (*x*_*i*_, *y*_*i*_), (*i*=1,2.., *n*). *x* ∈ *R*^*d*^, *y* ∈ {+1, −1}, and meet the conditions:

(8) $$\begin{array}{c} y_{i}\left[\left(w*x_{i}\right)+b\right]-1\geq 0,\;\left(i=1,2,\ldots ,n\right). \end{array}$$

Then, (*w*, *b*) defines a hyperplane, and the classification interval is equal to 2/‖*w*‖^2^. Make the maximum interval equal to the minimum 1/2‖*w*‖^2^, and satisfy constraint condition formula equation (8), which embodies the idea of the SVM maximum interval.

The Lagrange function is constructed:

(9) $$\begin{array}{c} L\left(w,a,b\right)=\frac{1}{2}{\left\|w\right\|}^{2}-\sum_{1}^{n}a_{i}y_{i}\left(wx+b\right)+\sum_{1}^{n}a_{i}. \end{array}$$

The partial differentiation of *w* and *b* is obtained, respectively. The original problem is transformed into a dual problem:

(10) $$\begin{array}{c} \text{maximize }W\left(a\right)=\overset{N}{\underset{i=1}{\sum }}a_{i}-\frac{1}{2}\overset{n}{\underset{i=1}{\sum }}\overset{n}{\underset{j=1}{\sum }}a_{i}a_{j}y_{i}y_{j}\left(x_{i}x_{j}\right)\\ \text{subject to }a_{i}\geq 0,\left(i=1,2,\ldots ,n\right),\overset{n}{\underset{i=1}{\sum }}y_{i}a_{i}=0. \end{array}$$

After solving *a*, we use the weight vector *W*_0_(*W*_0_=∑_*i*_^*n*^*x*_*i*_*y*_*i*_*a*_*i*_(*i* ∈ support vector)) and *b*_0_ to determine the optimal hyperplane:

(11) $$\begin{array}{c} f\left(x\right)=\text{sign}\left[\sum_{i\in n}y_{i}a_{i}\left(x,x_{i}\right)+b_{0}\right]. \end{array}$$

For the nonlinear classification, the kernel function *K*(*x*_*i*_, *x*_*j*_) can be used to transform formula equation (10) into

(12) $$\begin{array}{c} W\left(a\right)=\sum_{i=1}^{N}a_{i}-\frac{1}{2}\sum_{i=1}^{n}\sum_{j=1}^{n}a_{i}a_{j}y_{i}y_{j}K\left(x_{i},x_{j}\right). \end{array}$$

Then, the corresponding classification function is

(13) $$\begin{array}{c} f\left(x\right)=\text{sign}\left[\overset{n}{\underset{i=1}{\sum }}y_{i}a_{i}K\left(x_{i},x_{j}\right)+b_{0}\right],\;\left(i\in \text{support vector}\right). \end{array}$$

Considering that some samples may not be classified correctly, in order to ensure the correctness of classification, the relaxation factor *ξ*_*i*_ ≥ 0(*i*=1,2, ···, *n*) is introduced, and constraint equation (8) becomes

(14) $$\begin{array}{c} y_{i}\left[\left(w*x_{i}\right)+b\right]-1+\xi_{i}\geq 0,\;\left(i=1,2,\ldots ,n\right). \end{array}$$

The optimization objective function becomes

(15) $$\begin{array}{c} \phi \left(w\right)=\frac{1}{2}\left(w*w\right)+C\sum_{i=1}^{n}\xi_{i}, \end{array}$$

where *C* is a specified constant, which is called error penalty factor. It plays a role in controlling the degree of penalty for wrong samples and achieves a compromise between the proportion of wrong samples and the complexity of the algorithm.

At present, there are four kinds of kernel functions that are widely used:

(i) Linear kernel:
  (16) $$\begin{array}{c} K\left(u,v\right)=\left(u,v\right). \end{array}$$
(ii) Poly kernel:
  (17) $$\begin{array}{c} K\left(u,v\right)={\left(r\left(u,v\right)+\text{coef}0\right)}^{d}. \end{array}$$
(iii) RBF kernel:
  (18) $$\begin{array}{c} K\left(u,v\right)=\exp\left(-r{\left|u-v\right|}^{2}\right). \end{array}$$
(iv) Sigmoid kernel:

(19) $$\begin{array}{c} K\left(u,v\right)=\tanh\left(r\left(u,v\right)+\text{coef}0\right). \end{array}$$

An important step is to select kernel function and kernel parameter when the SVM is used in weather forecast. Vapnik et al. found that different kernel functions have little effect on the SVM performance, but the parameters of kernel function and error penalty factor *C* are the key factors affecting the SVM performance. It is very important for the performance of learning machine to choose a proper kernel parameter and error penalty factor *C*. At present, there is not a scientific theory to solve the optimization problem of SVM classifier model parameters. It is still in the stage of repeated experiments trying to choose the optimal kernel function and related parameters for the experimental results. Therefore, in order to improve the recognition rate and speed, this paper tests the performance of the above four kernel functions on the weather dataset at the same time in order to find the optimal model.

## 3. IQGA-SVM Model

Support vector machines have good generalization ability. They can solve the problems of small sample and nonlinearity and overcome the shortcomings of traditional methods, such as dimension disaster and overlearning. However, there are many aspects that affect the recognition accuracy of the SVM including the selection of kernel function which is not only often related to human experience but also to sample data. It is impossible for human experience to be effective for all sample data. There are many optimization methods for parameter variables in practical problems, among which search algorithm is widely used. It searches for the whole parameter variable space. It is necessary to select an effective search algorithm to obtain the optimal parameters considering the large parameter space and the limited calculation time. Aiming at the weather prediction system studied in this paper, the parameter optimization of the traditional SVM classification model has reached the goal of improving the classification accuracy. Based on this, the IQGA-SVM classification model is constructed. The optimization ability of the improved quantum genetic algorithm is discussed in the following.

The quantum genetic algorithm starts from the initial population which is composed of the whole solution space with average distribution to search in the parameter space. The individual state is represented by the quantum bit probability amplitude, which enhances the diversity of the population and expands the optimization ability. Individuals with higher adaptability are selected for population renewal through the selection operation in genetic operation. Population renewal is carried out through the quantum rotation gate. Population renewal means that there are always new individual transformations which not only enhance the population diversity but also expand the scope of search and can obtain the global optimal solution better than genetic algorithm. Quantum genetic algorithm only needs to calculate the fitness value. Therefore, it is convenient to connect the SVM as an intermediate variable for parameter optimization.

**Figure 6:**
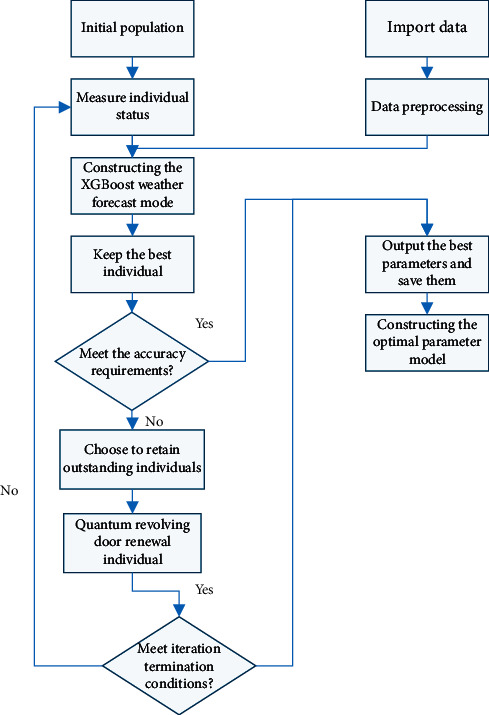
IQGA-SVM algorithm flow.

IQGA-SVM algorithm flow is as follows:

- First, a predesigned angle sequence is generated by initialization. Then, it is transformed into a population of *n* individuals *P*(*t*)(*P*(*t*)=(*p*_1_^*t*^, *p*_2_^*t*^,…, *p*_*n*_^*t*^)), where *p*_*j*_^*t*^(*j*=1,2,…, *n*) is an individual of generation *t* in the population.
- Construct *R*(*t*)=(*a*_1_^*t*^, *a*_2_^*t*^,…, *a*_*n*_^*t*^) according to the value of the probability amplitude in *P*(*t*), where *a*_*j*_^*t*^(*j*=1,2,…, *n*) is a binary of string.
- Use the fitness function to calculate each individual in *R*(*T*). Next, use the following selection strategy. The fitness is used to determine the probability that the individual is left behind, so as to ensure that each individual can have the probability corresponding to the fitness and keep it. This method can not only keep the population rich and avoid falling into the local optimal solution but also make the whole population converge in a better direction.
- Judge whether the specified precision termination condition is triggered. If not, continue the following operations. If the condition is met, output the optimal parameter and construct the optimal parameter model.
- According to the number of iterations, the rotation angle of the quantum rotation gate is changed dynamically.
- Determine whether the set upper limit of iteration times is reached. If the upper limit is reached, output the optimal parameters, and construct the optimal parameter model. The algorithm flow of the weather prediction model based on IQGA-SVM is shown in Figure 6.

## 4. Simulation

In order to verify the feasibility of the model, the partial random sampling of the weather dataset is carried out, and the simulation experiment is carried out on software Jupyter Notebook, and Python 3.7 version is used for testing.

**Figure 7:**
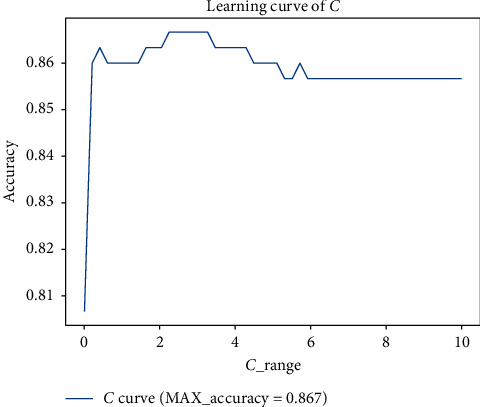
Learning curve of linear kernel function *C*.

**Figure 8:**
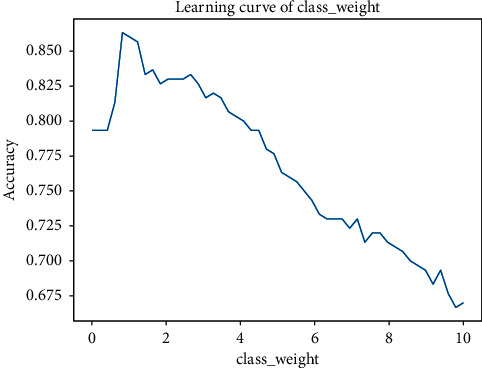
Learning curve of linear kernel function class weight.

In order to reduce the search range of the improved quantum genetic algorithm, the general range of the parameters to be adjusted is determined by the traditional learning curve of parameter adjustment. We found that the tag of the weather dataset has the problem of sample imbalance after data exploration. The proportion of rainy weather and nonrainy weather is about 3 : 1, so we need to add a class_weight balance parameter when building the model. As shown in Figures 7 and 8, Parameters *C* and class_Weignt have great influence on the performance of the algorithm.

Through observation, it can be found that, for IQGA-SVM, the model of the weather forecast dataset in this study, the penalty coefficient *C* is better in [0,3], and the sample unbalanced parameter class_weight is the best in [0.5, 2]. Through the exploration of different parameter ranges of the four kernel functions by the learning curve and grid search method, the final parameters and ranges to be found are shown in Table 3. After a lot of experiments, the parameter settings of the improved quantum genetic algorithm are shown in Table 3.

**Figure 9:**
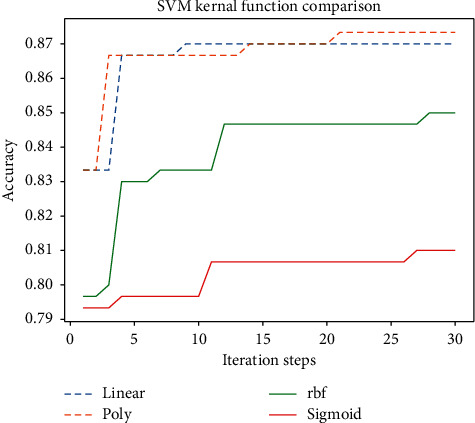
SVM kernel function comparison.

After determining the range of the parameters to be searched, the improved quantum genetic algorithm is used to optimize the parameters of the four kernel functions, respectively, and the optimization effect is shown in Figure 9.

Through the analysis of Figure 9, it can be concluded that the prediction effect of the linear kernel function and poly kernel function on the weather dataset used in this study is the best. Among them, the highest prediction accuracy of the model using the poly kernel function is 87.33%, and the highest prediction accuracy of the model using the linear kernel function is 87%. Moreover, the improved quantum genetic algorithm can find the optimal parameter value for the model in a few iterations. The effect of the rbf kernel function is slightly lower than the former two, and its highest accuracy is 85.3%. The performance of the sigmoid kernel function is the worst. The highest prediction accuracy is only 81.3%. Therefore, it can be seen that this weather dataset is a partial linear dataset through analysis. The optimal parameter list determined by improving the optimization of quantum genetic algorithm is shown in Table 4.

**Figure 10:**
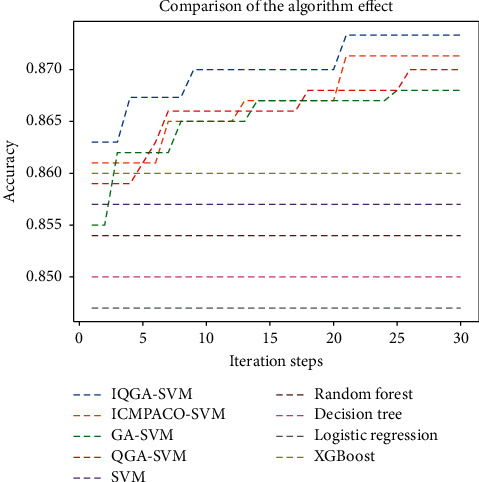
Comparison of the algorithm effect.

**Figure 11:**
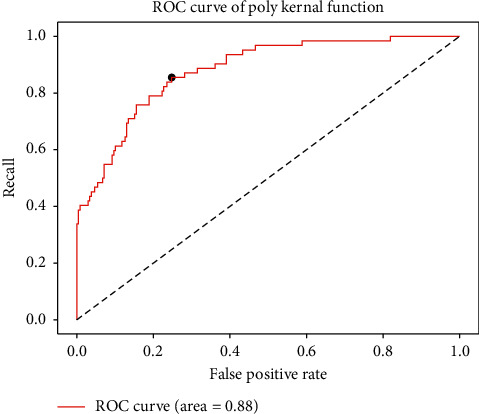
ROC curve of the poly kernel function.

Next, IQGA-SVM model (poly kernel function), support vector machine model based on genetic algorithm (GA-SVM), and support vector machine model based on improved ant colony optimization (ICMPACO-SVM) [39] are tested for 30 times, respectively, and compared with other machine learning models. The results are shown in Figure 10. The performance comparison results of each algorithm are shown in Table 5. As can be seen from Table 5, the prediction accuracy of the SVM is better than that of linear regression, decision tree, and random forest and slightly lower than that of XGBoost. Genetic algorithm effectively improves the prediction accuracy of the SVM and surpasses other traditional machine learning algorithms. Quantum genetic algorithm is better than genetic algorithm but lower than ICMPACO-SVM and IQGA-SVM. The prediction rate of the model optimized by ICMPACO algorithm is 87.1%, second only to IQGA-SVM. The average accuracy, optimal accuracy, and AUC of the IQGA-SVM model are the highest, and the interpretability is also enhanced. At the same time, we also find that IQGA-SVM can converge to a better solution in a very short time, but at the end of the iteration, the search scope will gradually become smaller and occasionally fall into the local optimal solution. The ROC curve of the optimal IQGA-SVM model is shown in Figure 11.

Next, we introduce the parameter setting of several other parameter searching algorithms:

1. Grid search: the parameters include *C*=[0,3], *γ*=[0,3], Cofe0=[0,10], and class_weight=[0.5, 2]. Use a 10*∗*10*∗*10*∗*10 grid to search.
2. Random walk: the parameters include steps = 0.5, precision control (epsilon = 0.00001), number of variables (var_num = 4), initial point (*X*[1.5, 1.5, 5,1]), and iter_num = 100.
3. GA: parameter search scope is the same as grid search. Population size *P* = 200, coding length *L* = 24, probability retention method, multipoint crossover, crossover probability Pc = 0.8, basic bit variation, mutation probability Pm = 0.005, and the termination condition is to meet the corresponding accuracy. Number of iterations *g* = 100.
4. ICMPACO: parameter search scope is the same as grid search. Ant number = 50, pheromone factor *α*=1, heuristic factor *β*=4, volatility coefficient *ρ*=0.1, pheromone amount *Q* = 100, initial concentration *τ*_*ij*_(0)=1.5, and maximum iterations *T* = 200.

The IQGA-SVM is compared with the commonly used parameter adjustment methods to verify its advantages in accuracy and efficiency. Grid search is the most commonly used method of parameter adjustment in the industry. Its idea is exhaustive search, traversing all the candidate parameters to find the best value of the parameters to be selected. Its disadvantage is that it takes a long time and can only be selected in the given candidate set. Random walk is a global optimization algorithm. According to the law of large numbers, as long as the random number is enough, the optimal or near-optimal parameters can always be found. Its disadvantage is that it highly depends on the initial value, and the random results may be inconsistent. The idea of quantum genetic algorithm is the survival of the fittest. Keep some excellent individuals in each iteration, and there is a certain probability of crossover and mutation, resulting in new samples. Therefore, quantum genetic algorithm is also a global optimization algorithm. When the mutation probability is large, quantum genetic algorithm will degenerate into random walk. IQGA-SVM, QGA-SVM, GA-SVM, grid search, and random walk were used to adjust parameters for 30 times, respectively. The comparison results are shown in Table 6. The order of parameter list is *C*, *γ*, coef0, degree, and class_weight.

Table 5 uses average accuracy, optimal accuracy, and running time as evaluation indexes. From Table 5, it can be found that grid search accuracy is low and extremely time consuming. Random walk accuracy and running time are in the middle. The running time of GA-SVM is the least, and its precision is second only to QGA-SVM. The running time of ICMPACO is longer than that of random walk, but its precision is second only to IQGA. IQGA has the best prediction accuracy, and the time is slightly higher than GA-SVM and QGA-SVM.

The experimental results show that IQGA-SVM is better than linear regression and random forest algorithm in prediction accuracy. It is better than grid search and random walk in running time and parameter adjustment. Compared with the GA-SVM algorithm, IQGA-SVM can improve the prediction accuracy at a sacrifice of a small amount of time.

## 5. Conclusion

In this paper, the real complex weather dataset is selected as the experimental data through ingenious data preprocessing to enhance the potential of data mining. Then, the traditional quantum genetic algorithm is improved from several directions which effectively enhances the global search ability and efficiency of the quantum genetic algorithm. Next, the effects of IQGA-SVM, QGA-SVM, GA-SVM, and other classical machine learning algorithms on the dataset are compared. At the same time, IQGA-SVM is compared with grid search, random walk, QGA-SVM, and GA-SVM model in a variety of evaluation indicators. The advantages of the improved quantum genetic algorithm in parameter optimization are verified from several aspects. The experiment shows that the model of support vector machine based on the improved quantum genetic algorithm established in this paper has the optimal prediction effect. Through data analysis, the improved quantum genetic algorithm effectively improves the performance of the classifier and the accuracy of the weather prediction system by optimizing parameters. At the same time, we also find that the search range of IQGA-SVM will gradually decrease in the late iteration and occasionally fall into the local optimal solution. This leads to poor optimization effect. Our analysis shows that it may be because a large number of individuals are concentrated in the local optimal solution and cannot jump out at the end of the iteration. In the next study, we will consider adding the dynamic catastrophe strategy to solve this problem and further improve the optimization effect. This study has good academic research and engineering application value. The main contribution of this paper is to propose a new parameter optimization method. In the next research, we will further optimize the effect of quantum genetic algorithm. At the same time, it is combined with more machine learning algorithms to increase its scalability. It can improve the efficiency and accuracy of optimization by using the parallelism and good global search ability of quantum algorithm. It can also be applied to the weight and bias optimization of the neural network, so as to improve the learning ability of the neural network.

## Figures and Tables

**Table 1:** Partial weather dataset.

Date	Location	MinTemp	MaxTemp	Rainfall	Sunshine u̇u̇	Humidity	RainTomorrow
2014/8/26	Launceston	3.5	18.4	0	2.5	66	No
2013/5/1	Perth	14.4	25.7	0	Null	38	Yes
2017/5/9	Wagga	1.4	19	0	0.6	32	No
2016/6/11	Uluru	7.2	17.4	0	0.8	40	No
2010/3/26	Cairns	23.2	28.3	4.4	1.3	93	Yes

**Table 2:** New quantum rotation gate strategy.

*x*_*i*_	*b*_*i*_	*f*(*x*) ≥ *f*(best)	Δ*θ*	*s*(*α*_*i*_, *β*_*i*_)			
				*α*_*i*_ *β*_*i*_ > 0	*α*_*i*_ *β*_*i*_ < 0	*α*_*i*_=0	*β*_*i*_=0
0	0	False	*θ*__new_	−1	+1	±1	0
0	0	True	*θ*__new_	−1	+1	±1	0
0	1	False	*θ*__new_	+1	−1	0	+1
0	1	True	*θ*__new_	−1	+1	±1	0
1	0	False	*θ*__new_	−1	+1	±1	0
1	0	True	*θ*__new_	+1	−1	0	±1
1	1	False	*θ*__new_	+1	−1	0	±1
1	1	True	*θ*__new_	+1	−1	0	±1

**Table 3:** IQGA parameter and kernel function to be found parameter range.

IQGA parameter value			SVM value of kernel function			
Parameter	Value		Kernel function			
Population number	100	Parameter	Linear	Poly	rbf	Sigmoid
Chromosome number	4	*γ*	No	[0,3]	[0,3]	[0,3]
Chromosome length	20	*d*	No	1	No	No
*θ*_max	0.005*π*	Cofe0	No	[0,10]	No	[0,10]
iter_num	30	*C*	[0,3]	[0,3]	[0,3]	[0,3]
		class_weight	[0.5, 2]	[0.5, 2]	[0.5, 2]	[0.5, 2]

**Table 4:** Search result table.

Kernel	*C*	*γ*	Cofe0	*d*	class_weight
Linear	0.4759025573730469	No	No	No	0.8845815658569336
Poly	1.9044914245605469	0.0014705657958984375	8.277586	1	1.908930778503418
rbf	2.34393310546875	0.00496673583984375	No	No	1.8635177612304688
Sigmoid	0.7698040008544922	0.0029439926147460938	6.24142	No	1.2771968841552734

**Table 5:** Algorithm performance comparison.

Model	Average accuracy (%)	Optimal accuracy (%)	AUC
Random forest	—	85.4	0.85
Decision tree	—	85.0	0.85
Logistic regression	—	84.7	0.84
XGBoost	—	86.0	0.86
SVM	—	85.7	0.85
GA_SVM	86.5	86.7	0.87
QGA_SVM	86.7	87.0	0.86
ICMPACO_SVM	86.8	87.1	0.88
IQGA_SVM	87.1	87.3	0.88

**Table 6:** Comparison of parameter adjustment methods.

Method	Average accuracy (%)	Optimal accuracy (%)	AUC	Running time (s)	Parameter list
Grid search	85.3	85.8	0.862	3056	[2.23, 0.05, 3.3, 1, 1.3]
Random walk	85.5	86.2	0.866	1827	[1.78, 0.007, 6.53, 1, 1.56]
GA-SVM	86.5	86.7	0.870	1235	[2.19, 0.002, 7.23, 1, 1.91]
QGA-SVM	86.7	87.0	0.870	1302	[1.97, 0.005, 8.84, 1, 1.51]
ICMPACO-SVM	86.8	87.1	0.880	2389	[1.823, 0.0008, 8.5, 1, 1.45]
IQGA-SVM	87.1	87.3	0.880	1364	[1.90, 0.001, 8.3, 1, 1.56]

## Data Availability

The data used in this article are available at https://www.kaggle.com/jsphyg/weather-dataset-rattle-package.
