# Corrigendum to “Support Vector Machine Weather Prediction Technology Based on the Improved Quantum Optimization Algorithm”

**DOI:** 10.1155/2022/9762403

**Published:** 2022-02-10

**Authors:** Jinlei Zhang, Xue Qiu, Xiang Li, Zhijie Huang, Mingqiu Wu, Yumin Dong

**Affiliations:** College of Computer and Information Science, Chongqing Normal University, Chongqing, China

## Abstract

As we all know, the weather forecast is very complex, which is related to the location of the environment, climate, season, and other factors. The accuracy of weather prediction is of great significance for the application of hydrological and agroclimatological research. In this research, the improved quantum genetic algorithm (IQGA) and support vector machine (SVM) are combined to model the rainfall of medium-sized cities in Australia. The daily time scale measured weather information, such as min temperature (MT), evaporation, sunshine, humidity, cloud, and so on, which was used to build the proposed predictive models. Next, the effects of IQGA-SVM, GA-SVM, and other classical machine learning algorithms on the dataset are compared. Experiments show that the IQGA-SVM model has the optimal prediction ability and less running time. Statistical evaluation metrics such as accuracy, area under curve (AUC), and running time were used to validate the model efficiency. The IQGA-SVM model uses IQGA to optimize the parameters of SVM. Compared with the traditional random walk and grid search method, it improves the calculation efficiency and the accuracy of parameter optimization. This parameter optimization method has good expansibility and can be applied to other algorithms that need parameter adjustment to achieve the optimal model prediction effect. The results of this study proved that IQGA-SVM is a reliable modeling technique for forecasting rainfall.

In the article titled “Support Vector Machine Weather Prediction Technology Based on the Improved Quantum Optimization Algorithm” [1], the abstract is incorrect due to an error during the preparation of the manuscript and the correct abstract is above.
